# Systematic genome-wide Mendelian randomization reveals the causal links between miRNAs and Parkinson’s disease

**DOI:** 10.3389/fnins.2024.1385675

**Published:** 2024-05-03

**Authors:** Guolin Shi, Tingting Wu, Xuetao Li, Debin Zhao, Qiuyuan Yin, Lei Zhu

**Affiliations:** ^1^Department of Neurosurgery, The Second Affiliated Hospital of Kunming Medical University, Kunming, Yunnan, China; ^2^State Key Laboratory for Conservation and Utilization of Bio-Resources in Yunnan, School of Life Sciences, Yunnan University, Kunming, Yunnan, China

**Keywords:** Parkinson’s disease, miRNAs, Mendelian randomization, causal effect, biomarker

## Abstract

**Background:**

MicroRNAs (miRNAs) have pivotal roles in gene regulation. Circulating miRNAs have been developed as novel candidate non-invasive biomarkers for diagnosis, prognosis, and treatment response for diseases. However, miRNAs that have causal effects on Parkinson’s Disease (PD) remain largely unknown. To investigate the causal relationships between miRNAs and PD, here we conduct a Mendelian randomization (MR) study.

**Methods:**

This study utilized the summary-level data of respective genome-wide association studies (GWAS) for 2083 miRNAs and seven PD-related outcomes to comprehensively reveal the causal associations between the circulating miRNAs and PD. Two-sample MR design was deployed and the causal effects were estimated with inverse variance weighted, MR-Egger, and weighted median. Comprehensively sensitive analyses were followed, including Cochran’s *Q* test, MR-Egger intercept test, MR-PRESSO, and leave-one-out analysis, to validate the robustness of our results. Finally, we investigated the potential role of the MR significant miRNAs by predicting their target genes and functional enrichment analysis.

**Results:**

Inverse variance weighted estimates suggested that two miRNAs, miR-205-5p (β = −0.46, 95%CI: −0.690 to −0.229, *p* = 9.3 × 10^−5^) and miR-6800-5p (β = −0.389, 95%CI: −0.575 to −0.202, *p* = 4.32 × 10^−5^), significantly decreased the rate of cognitive decline among PD patients. In addition, eight miRNAs were nominally associated with more than three PD-related outcomes each. No significant heterogeneity of instrumental variables or horizontal pleiotropy was found. Gene Ontology (GO) analysis showed that the targets of these causal miRNAs were significantly enriched in cell cycle, apoptotic, and aging pathways.

**Conclusion:**

This MR study identified two miRNAs whose genetically regulated expression might have a causal role in the development of PD dementia. Our findings provided potential miRNA biomarkers to make better and early diagnoses and risk assessments of PD.

## Introduction

Parkinson’s disease (PD) is the second most common neurodegenerative disease with a global prevalence of more than 6 million individuals. This number has been projected to double over the next generation, making PD one of the leading causes of neurological disability (Dorsey et al., 2018; Feigin et al., 2019). Its main neuropathological hallmarks are the degeneration of dopaminergic neurons in the substantia nigra and alpha-synuclein-containing protein inclusions, called Lewy Bodies (Poewe et al., 2017). Due to the lack of a reliable objective biomarker, the diagnosis of idiopathic PD is still based on the assessment of clinical criteria, leading to insufficient diagnostic accuracy, especially in the early stages of the disease (Rizzo et al., 2016; Tolosa et al., 2021). Therefore, more biomarkers are needed to further enhance diagnostic accuracy and sensitivity for early or prodromal disease stages. Genome-wide association studies (GWAS) have reported multiple risk variants for PD (Nalls et al., 2019), and the vast majority of risk variants reside in the non-coding region, indicating the important roles of non-coding regions in the development of PD. However, most of the published integrative studies on PD focused on mRNA or proteins, potentially missing important biological functions of non-coding transcripts, such as miRNAs.

MiRNAs are small (about 22 nucleotides) non-coding RNAs involved in the regulation of gene expression (Bartel, 2018) and play key roles in different biological processes, including cell fate determination, embryonic development, cell proliferation, differentiation, and apoptosis (Satterlee et al., 2007). MiRNA is a useful biomarker in some pathologies, such as cancer (Jamali et al., 2018) and cardiovascular disease (de Gonzalo-Calvo et al., 2019). Mounting evidence has also shown that extracellular circulating miRNAs can be detected in biological fluids such as blood, urine, serum, plasma, and cerebrospinal fluid and have a proven high chemical stability (Chen et al., 2008; Gallo et al., 2012). Therefore, miRNAs have emerged as novel candidate non-invasive biomarkers for diagnosis, prognosis, and treatment response for diseases (Danborg et al., 2014). Considering the important roles of miRNAs in physiology and disease, some studies were performed to identify different expression miRNAs between PD patients and healthy control (Oliveira et al., 2020; Soto et al., 2023). Owing to the inherent defects of conventional designs, previous PD studies on miRNAs are unable to entirely exclude the possibility of reverse causality and confounding factors, which potentially results in biased associations and conclusions (Sekula et al., 2016). Besides, the results of previous studies usually implicate association, but not causality. These conditions limit the reliability of certain miRNAs as biomarkers of PD.

Mendelian Randomization (MR) is a powerful approach that uses genetic variants as instrumental variables (IVs) to estimate the causal effect of exposure on outcome (Richmond and Smith, 2022). Confounding bias can be minimized in MR studies because genetic variants are randomly assigned to the individual at birth. Similarly, reverse causation can be avoided because genetic variants are assigned before the development of the disease (Davey Smith and Hemani, 2014). MR has been widely used to explore the causality between miRNAs and diseases, such as COVID-19 (Li et al., 2021), schizophrenia (Mu et al., 2023), lung cancer (Huang et al., 2020), incident acute coronary syndrome (Shen et al., 2021), and type 2 diabetes (Mens et al., 2021).

In this study, to investigate the potential of miRNAs in predicting and treating PD cases, we adopted the summary statistics of miRNA expression quantitative trait loci (miR-eQTL) data for 2083 mature human miRNAs in blood samples as exposure and seven PD-related phenotypes as the outcome to explore the causal relationship between circulating miRNAs and PD. Identifying the causal mechanisms will be an important step in the diagnosis and treatment of PD.

## Materials and methods

### Study design

**Figure 1:**
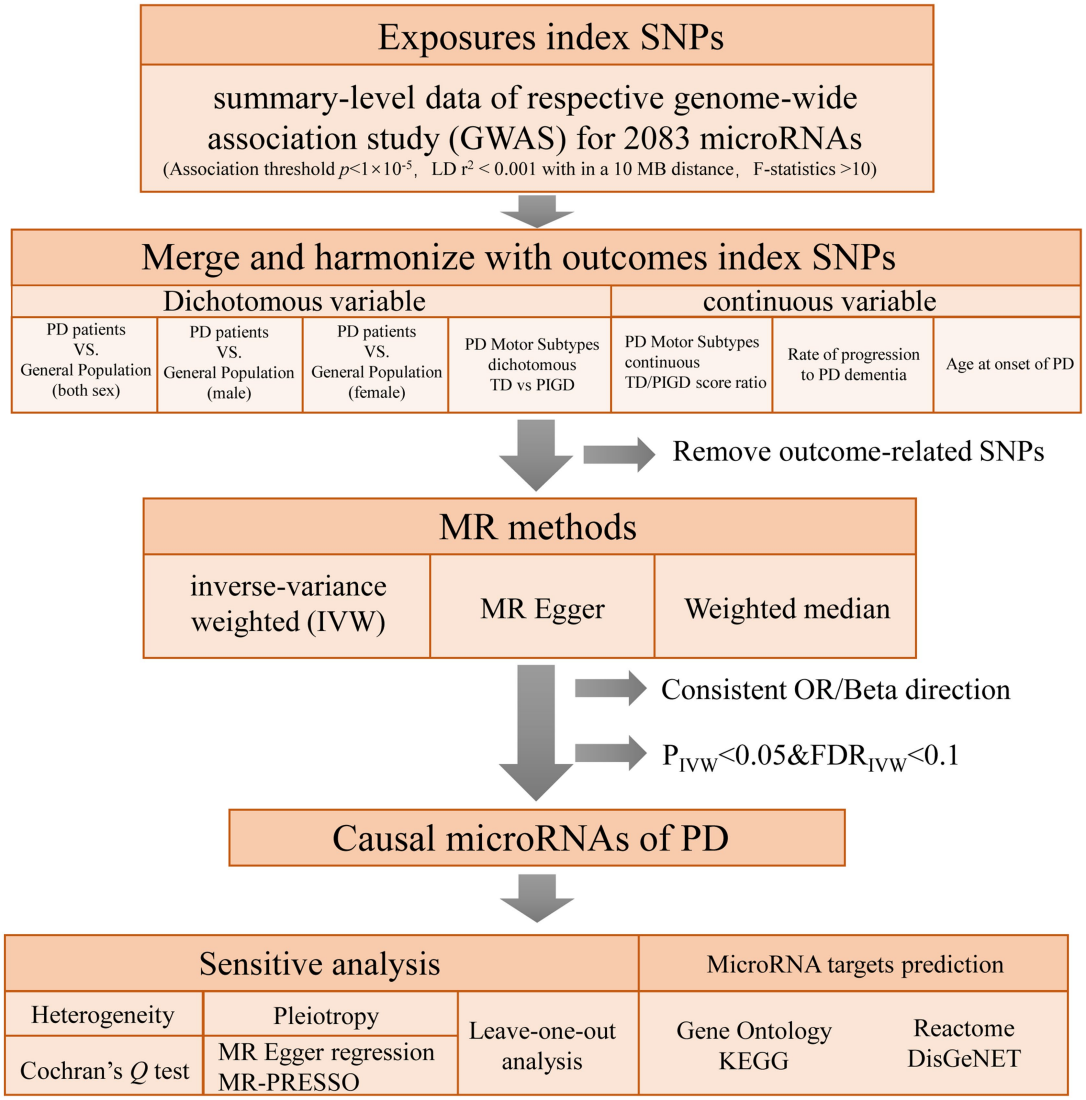
Flowchart of the study design. PD: Parkinson’s Disease; TD: tremor dominant; PIGD: postural instability/gait difficulty.

To explore the causal relationships of miRNAs on PD, a two-sample MR was performed using instrumental variables (IVs) extracted from the largest investigation of the genetics of miRNAs (Nikpay et al., 2019). seven PD-related phenotypes GWAS were used as outcome. MR design is based on three assumptions: (1) genetic variants are robustly associated with exposure data; (2) genetic variants are not associated with potential confounders; and (3) genetic variants affect the outcome only through the exposure of interest (Boef et al., 2015). In this study, multiple methods were used for MR and sensitivity analyses to confirm the robustness of our results. Enrichment analysis based on four databases was performed to investigate the potential role of the MR significant miRNAs, including Gene Ontology, KEGG, Reactome, and DisGeNET database. The conceptual framework of this study is presented in Figure 1.

### Exposure data

In this study, we used the largest miRNA expression quantitative loci (eQTLs) data to date as our exposure. We obtained the miRNA eQTL data from a recent study (Nikpay et al., 2019). This study comprehensively examined 2083 mature human miRNAs and their expression levels in blood samples among 710 unrelated people of European ancestry. Sex, age, first 10 principal components, and genotyping batch were corrected during the analysis.

### Outcome data

To study the causal effect of miRNAs on PD comprehensively, we collected GWAS summary statistics of seven PD-related phenotypes. We retrieved genetic data for Parkinson’s disease risk from the most recent GWAS meta-analysis of 16 cohorts from the International Parkinson’s Disease Genomics Consortium (IPDGC), with 37,688 cases, 18,618 proxy-cases (individuals without a diagnosis of PD but with a first degree relative with PD diagnosis), and 1,417,791 controls (Nalls et al., 2019). According to previous PD studies, age is the most significant risk factor for developing PD, and men are more susceptible than women with a prevalence ratio of approximately 3:2 (Blauwendraat et al., 2020). So we got the largest GWAS summary data of age at the onset of PD (*n* = 28,568) (Blauwendraat et al., 2019). We also obtain the GWAS summary statistics of PD in male and female patients from a recent study, which is consisting of 13,020 male PD cases, 7,936 paternal proxy cases, 89,660 male controls, 7,947 female PD cases, 5,473 maternal proxy cases, and 90,662 female controls (Blauwendraat et al., 2021). Since PD is mainly viewed as a motor disorder, we then retrieved genetic data of PD motor subtypes, including tremor dominant (TD) and postural instability/gait difficulty (PIGD) forms, which have implications for disease progression (*n* = 3,212) (Alfradique-Dunham et al., 2021). In addition to the motor disorders of PD, cognitive impairment and dementia are commonly seen in the later stages of PD, we thus retrieved the GWAS summary statistics of the rate of progression to PD dementia (*n* = 3,923) (Real et al., 2023).

### Selection criteria of instrumental variables

As the three assumptions stated in the design of this study, quality control was performed on single nucleotide polymorphisms (SNPs) to assure our results were robust. Similar to most current MR studies, the genome-wide significance threshold (*p* < 5 × 10^−8^) was selected to screen SNPs. Because a limited number of SNPs meet the criteria of genome-wide significance, we used SNPs with a more relaxed threshold (*p* < 1 × 10^−5^) as potential IVs of each miRNA. To ensure independence among IVs, we applied linkage disequilibrium clumping with a clumping window of 10 MB and *R*^2^ < 0.001 based on European ancestry reference data from the 1,000 Genomes Project. Meanwhile, to avoid bias owing to the employment of weak instruments, *F* statistics were calculated for each SNP to measure the statistical strength, and only strong IVs (*F*-statistics >10) for each of our exposure remained. Ambiguous and palindromic SNPs of which the effect cannot be correct in the harmonizing process were excluded. Since MR frequently generates false positives in the presence of genetic correlation between traits (O’Connor and Price, 2018; Reay et al., 2022), the SNPs associated with the outcome (PD) were removed. Finally, according to previous reports, coffee drinking, smoking, education, and physical activity may affect PD (Bloem et al., 2021). Therefore, we removed the instruments that were significantly associated with these confounders in the PhenoScanner V2 database^1^ (Kamat et al., 2019).

### Mendelian randomization analyses

If there were two or more IVs, three different methods of MR, random-effect inverse-variance weighted (IVW), MR Egger, and weighted median, were performed to estimate the causal effect of miRNAs on PD. IVW estimates were used as the main analysis, which combined the Wald ratio of each SNP on the outcome and obtained a pooled causal estimate. If horizontal pleiotropy was not present, the IVW results would be unbiased (Burgess et al., 2016). Meanwhile, MR-Egger and weighted median were used to improve the IVW estimates as they could provide more robust estimates in a broader set of scenarios, despite being less efficient (wider confidence interval). MR-Egger allows all genetic variants to have a pleiotropic effect but requires that the pleiotropic effects be independent of the variant-exposure association (Bowden et al., 2015). The weighted median method allows for the correct estimation of causal association when up to 50% of instrumental variables are invalid (Hartwig et al., 2017). If only one IVs were available, the Wald ratio method was used for MR analysis.

### Sensitivity analysis

Sensitivity analysis has been performed to detect the underlying pleiotropy and heterogeneity because they can seriously affect MR estimates. Cochran’s *Q* test was applied to detect heterogeneity (Greco M et al., 2015). There was no heterogeneity detected if the *p-*value of Cochran’s *Q* test was >0.05. The pleiotropic analysis was preliminarily judged by the intercept of MR Egger regression (*p* < 0.05 was considered as possible pleiotropy in IVs) (Burgess and Thompson, 2017). MR-Pleiotropy Residual Sum and Outlier methods (MR-PRESSO) were also used to assess and correct horizontal pleiotropy (Ong and MacGregor, 2019). Meanwhile, a Leave-one-out analysis was performed to evaluate whether the MR estimate was driven or biased by a single SNP. We also used a constrained maximum likelihood and model averaging-based MR method, called cML-MA, to control correlated and uncorrelated pleiotropic effects in this study (Xue et al., 2021).

### Statistical analysis

*F*-statistic was used to calculate the strength of IVs by the formula $F=\frac{R^{2}\times \left(N-1-K\right)}{\left(1-R^{2}\right)\times K}$, where *R*^2^ represents the proportion of variance in the exposure explained by the genetic variants, *N* represents sample size, and *K* represents the number of IVs (Staiger and Stock, 1994). To account for multiple testing in our primary analyses, false discovery rate (FDR) correction was performed by applied *q*-value procedure, with a false discovery rate of *q*-value <0.1 (Storey and Tibshirani, 2003). MiRNAs and PD were considered to have a nominal association when *p* < 0.05 but *q* ≥ 0.1.

All the analyses were performed by the Two-Sample MR package (version 0.5.6) (Hemani et al., 2017), MRcML package (Xue et al., 2021), and qvalue package (version 2.15.0), (Storey and Tibshirani, 2003) of the R program (version 4.2.1).

### MiRNA target prediction

To further explore the potential function of the identified miRNAs, we used MiRNet 2.0^2^ to predict the target genes of these miRNAs. Only experimentally validated target genes were considered as represented by the miRTarBase v8.0 database (Huang et al., 2020). The predicted target genes were further used as input for enrichment analysis based on four databases, including Gene Ontology, KEGG, Reactome, and DisGeNET database, to explore if the target genes were enriched in specific biological processes.

## Results

According to the selection criteria of IVs, a total of 23,261 SNPs were used as IVs for 2083 miRNAs. *F* statistics for these genetic instruments were all larger than the normally selected value of 10, indicating strong instruments. Details about the selected instrumental variables are shown in Supplementary Table S1.

To comprehensively study the causal effect of miRNAs on PD, we performed a two-sample MR analysis between 2083 miRNAs and seven PD-related outcomes (Figure 1).

**Figure 2:**
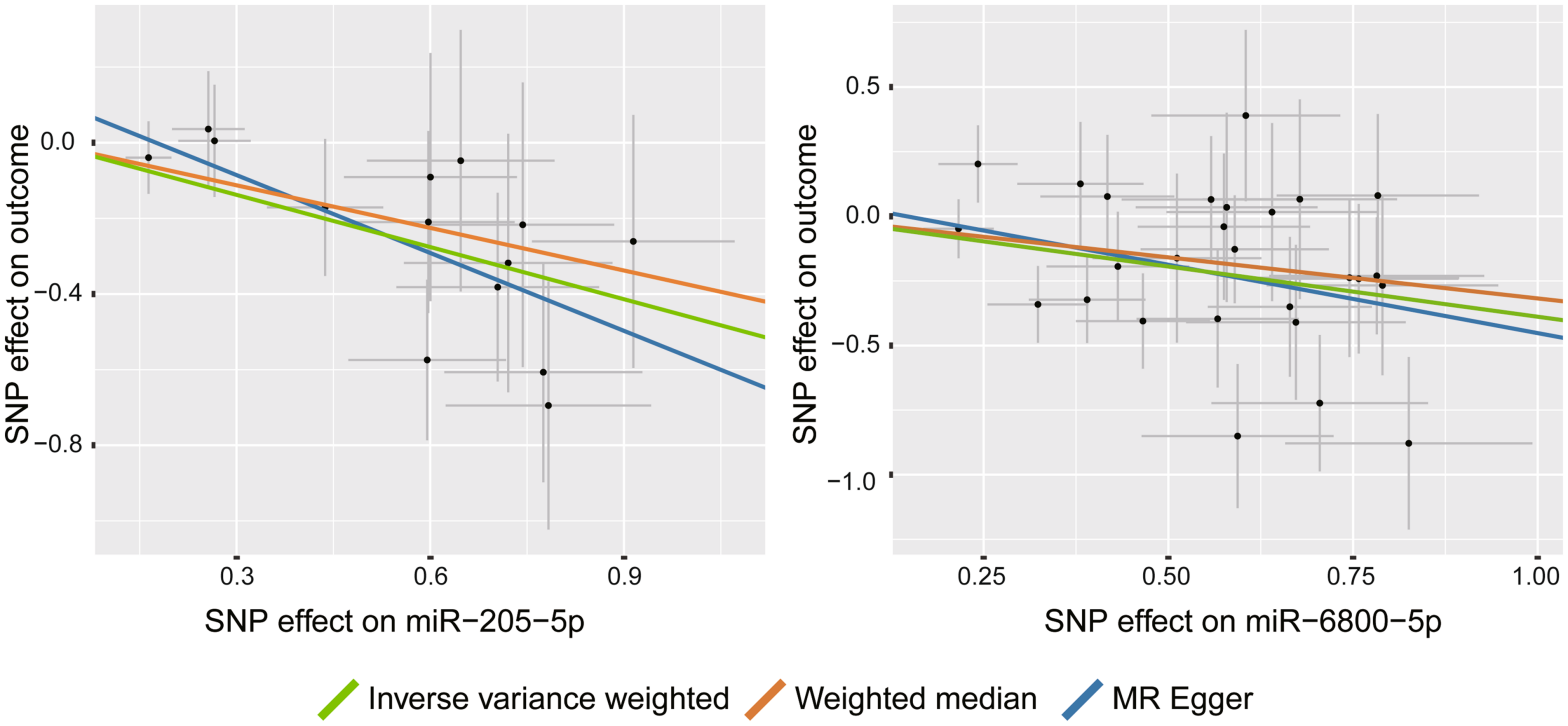
Scatter plots for the causal association between miRNAs and the rate of cognitive decline among PD patients.

Among the tested PD phenotypes, IVW analysis suggested that two miRNAs, miR-205-5p (β = −0.46, 95%CI: −0.690 to −0.229, *p* = 9.3 × 10^−5^) and miR-6800-5p (β = −0.389, 95%CI: −0.575 to −0.202, *p* = 4.32 × 10^−5^), significantly decreased the rate of cognitive decline among PD patients (Figure 2). The results from other MR methods showed a consistent results with nominal significance (Table 1), making our results more reliable. Our results suggested that miR-205-5p and miR-6800-5p had a protective effect on PD dementia.

**Table 1:** MR estimates for the causal relationship between miRNAs and the rate of progression to PD dementia.

MicroRNA (exposure)	MR method	No. of SNP	BETA	95%CI	*p*-value	*q*-value
miR-205-5p	IVW	14	−0.46	−0.690 – −0.229	9.30E-05	0.079
	MR Egger	14	−0.686	−1.138 – −0.234	0.012	0.997
	Weighted median	14	−0.375	−0.699 – −0.051	0.023	0.986
miR-6800-5p	IVW	27	−0.389	−0.575 – −0.202	4.32E-05	0.073
	MR Egger	27	−0.529	−1.032 – −0.027	0.05	0.997
	Weighted median	27	−0.318	−0.571 – −0.064	0.014	0.986

MR, Mendelian randomization, SNP, single nucleotide polymorphism, CI, confidence interval, IVW, inverse variance weighted.

**Figure 3:**
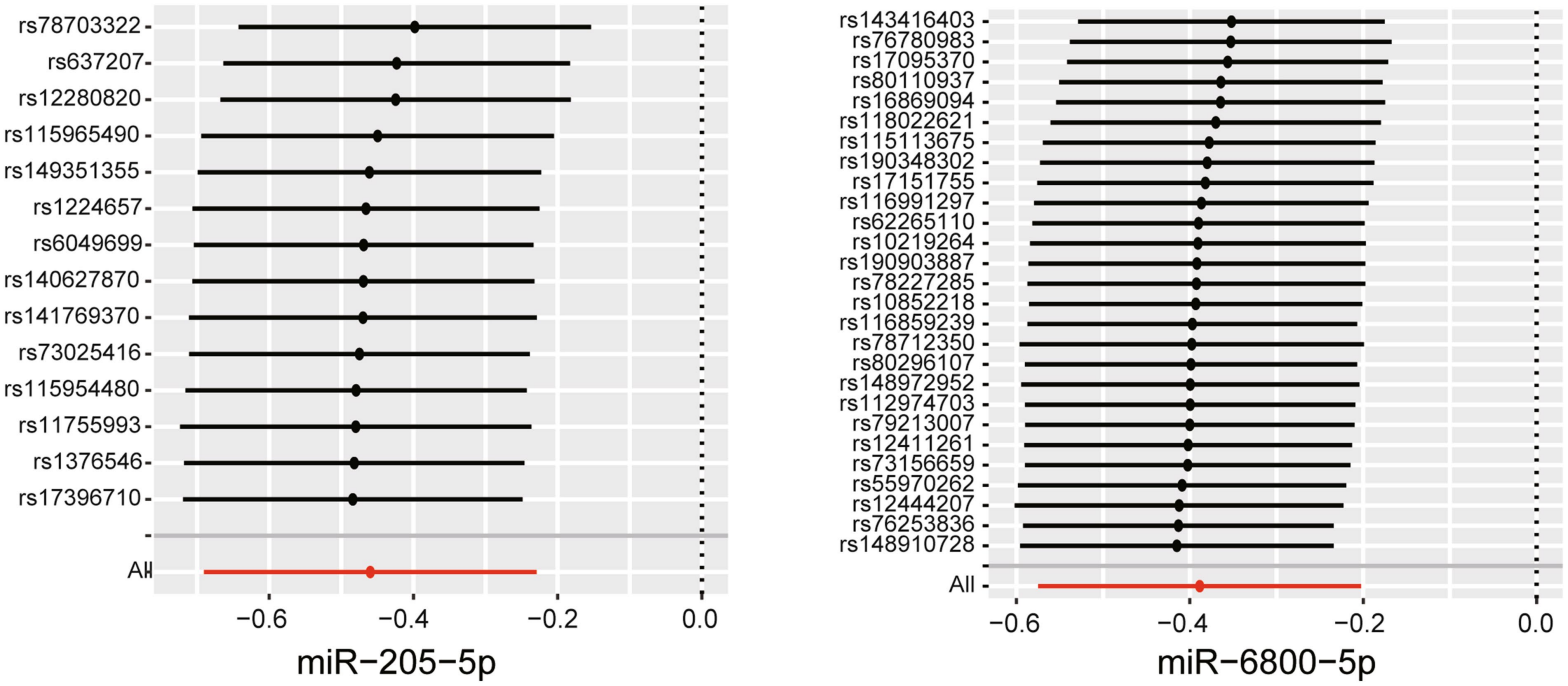
Leave-one-out plots for the causal association between miRNAs and the rate of cognitive decline among PD patients.

To assess the robustness of the above results, a series of sensitivity analyses, including Cochran’s Q test, MR Egger intercept test, and MR-PRESSO global test, were conducted. All *p* values of the above tests were >0.05, indicating that no heterogeneity and horizontal pleiotropy existed (Supplementary Tables S2–S4). To detect whether existed any high-influence SNPs biasing the MR results, a leave-one-out analysis was performed and the results showed there is no SNPs independently drove the results, indicating the reliability of our results (Figure 3). To further control correlated and uncorrelated pleiotropic effects in this study, the cML-MA-BIC method was used to recalculate the MR results of the two miRNAs. The results of cML-MA-BIC were consistent with IVW (Supplementary Table S5), which suggested our results were robust after considering the associated pleiotropy.

**Figure 4:**
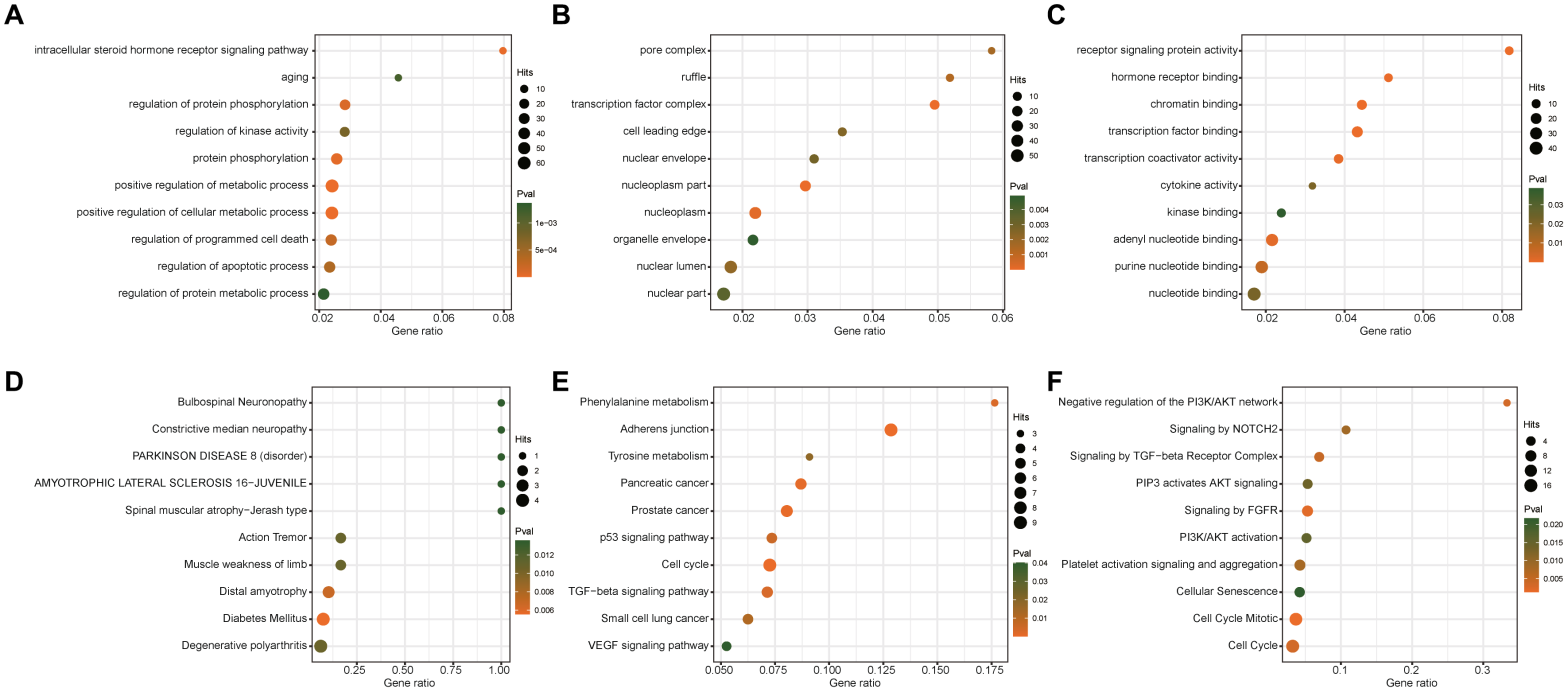
Enrichment analysis of targets of significant microRNAs. GO analysis results of biological process **(A)**, cellular component **(B)**, and molecular function **(C)**. **(D)** Enrichment analysis based on the DisGeNET database. **(E)** KEGG Enrichment analysis. **(F)** Reactome pathways analysis.

To further explore the potential roles of these significant miRNAs in PD dementia, we predicted the target genes of two causal miRNAs and performed enrichment analysis based on four databases, including Gene Ontology, KEGG, Reactome, and DisGeNET database. A total of 221 unique genes were identified to be regulated by these two miRNAs (Supplementary Table S6). Our results show that these target genes are significantly enriched in some previously reported PD-related pathways, such as metabolic process, cell cycle, aging, and protein phosphorylation (Figure 4).

**Figure 5:**
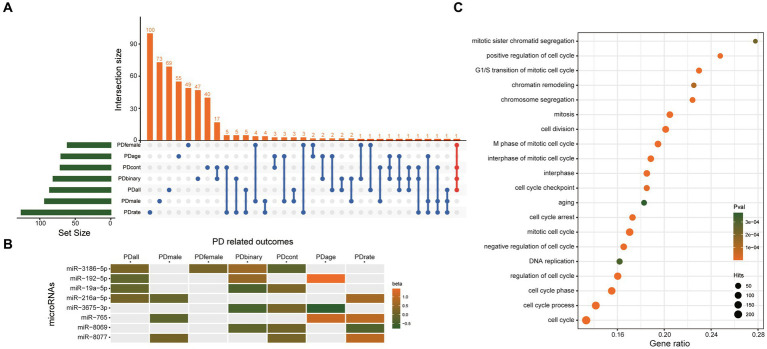
The eight miRNAs which were nominally associated with more than three PD-related outcomes. **(A)** UpSetR plot shows the overlap of miRNAs nominally associated with different PD-related outcomes. **(B)** Heatmap shows the beta value of the eight miRNAs which was nominally associated with more than three PD-related outcomes. **(C)** GO analysis results. Only the top 20 most significant pathways are shown. The target genes showed the most significant enrichment in the cell cycle. PDall means PD patients of both sexes. PDmale means male PD patients. PDfemale means female PD patients. PDage menas age at onset of PD. PDbinary means dichotomous motor subtype (TD vs. PIGD). PDcont means a continuous tremor/PIGD score ratio. PDrate means the rate of cognitive decline among PD patients.

Besides the two significant causal miRNAs above, 506 miRNAs were nominally associated with the seven PD-related outcomes (Supplementary Table S7). Although their q value is>0.1, which means they may be false positives, there must be real causal miRNAs of PD hiding in them. Therefore, we choose the miRNAs which were nominally associated with more than three PD-related outcomes as our candidate causal miRNAs of PD. Finally, eight miRNAs were found, including miR-3186-5p, miR-192-5p, miR-19a-5p, miR-216a-5p, miR-3675-3p, miR-765, miR-8069, miR-8077 (Figure 5A), and their beta value of IVW in different outcomes were presented in Figure 5B. A total of 1807 unique genes were predicted as potential targets of these eight miRNAs (Supplementary Table S8). GO analysis (using a biological process as GO term) showed that the target genes of these eight miRNAs were significantly enriched in the cell cycle (Figure 5C). These results suggested that these eight miRNAs may play an important role in the regulation of cell cycle through their target genes.

## Discussion

We performed the first genome-wide MR study to investigate the causal relationships between miRNAs and PD. We identified two MR-significant miRNAs that may have causal effects on the rate of cognitive decline among PD patients and eight miRNAs nominally associated with more than three PD-related outcomes. In addition, the target genes of the above miRNAs were significantly enriched in PD-related pathways, which supported the important role of these miRNAs in PD.

Although PD is mainly viewed as a motor disorder, the onset of dementia within PD significantly impacts morbidity, mortality, and social support needs (Szeto et al., 2020). The clinical and pathological characteristics of PD dementia often resemble dementia with Lewy bodies. However, in PD dementia, motor symptoms precede dementia by at least one year (Dubois et al., 2007). PD dementia is marked by neuropsychiatric symptoms, including cognitive fluctuations, visual misperceptions, hallucinations, and delusions, alongside deficits in attention, executive function, and visuo-spatial abilities. However, there is currently no cure for the underlying pathology of PD dementia. Therefore, elucidating the causal links between miRNAs and PD dementia is essential for developing novel treatments (Szeto et al., 2020; Real et al., 2023). Our study found that miR-205-5p and miR-6800-5p have a significant protective effect on PD dementia. Previous studies have shown that miR-205-5p directly regulates the expression of leucine-rich repeat kinase (LRRK2) (Chen et al., 2018), which was proven to contribute to the etiology of sporadic PD (Zimprich et al., 2004; Lin et al., 2009; Tolosa et al., 2020). Quantitative Real-Time PCR (qRT-PCR) and western blot assay showed that the expression levels of LRRK2 were increased, and the miR-205-5p level was decreased in the midbrains of PD mice (Chen et al., 2018), which suggested that downregulated miR-205 probably contribute to the increased LRRK2 protein level in the brains of patients with sporadic PD. Another similar research proposed that overexpression of miR-205 may provide an applicable therapeutic strategy to suppress the abnormal upregulation of LRRK2 protein in PD (Cho et al., 2013), which was consistent with our findings. Additionally, the methylation research of miR-205 promoter also support our results. There is an observed increase in methylation of the miR-205 promoter region in cells of the PD model. The findings from the methylation inhibition assay demonstrate that hypomethylation of the miR-205 promoter region effectively suppresses LRRK2 expression (Wang et al., 2022). MiR-6800 level was significant higher in L2NMC (LRRK2 non-manifesting carriers) than in L2PD (LRRK2 carrier with symptomatic PD) (Soto et al., 2023), which implies that miR-6800 have a significantly protective effect on PD. LRRK2 is a pivotal factor not only in Parkinson’s disease but also in the pathogenesis of dementia with Lewy bodies (Zhu et al., 2006). These previous studies implied that miR-205-5p and miR-6800-5p may slow down the rate of cognitive decline among PD patients by regulating expression of LRRK2.

The target genes of the two causal miRNAs are significantly enriched in some previously reported PD-related pathways. For example, Protein phosphorylation plays a crucial role in the pathophysiology of PD, particularly by influencing the phosphorylation of α-synuclein to promote its aggregation and toxicity, which are significant factors in neuronal damage and death in PD (Braithwaite et al., 2012). Additionally, phosphorylation regulates dopamine signaling pathways, mitochondrial function, and related cellular signaling cascades, with aberrations potentially exacerbating PD progression (Bohush et al., 2018). Cell cycle and aging also play an important role in PD pathogenesis. Alterations in cell cycle protein expression, and acceleration of aging processes may exacerbate neuronal damage and death, thereby contributing to PD progression. Conversely, PD itself may impact metabolic regulation, cell cycle dynamics, and aging processes, forming a vicious cycle (Joseph et al., 2020; Tchekalarova and Tzoneva, 2023). The above results further confirmed the relationship between miR-205-5p, miR-6800-5p and PD.

We also found eight miRNAs nominally associated with more than three PD-related outcomes, including miR-3186-5p, miR-192-5p, miR-19a-5p, miR-216a-5p, miR-3675-3p, miR-765, miR-8069, and miR-8077. Numerous studies have indicated that miR-192-5p plays a pivotal role in regulating oxidative stress, cellular proliferation, apoptosis, and inflammatory responses. Moreover, its association with various nervous system disorders such as Alzheimer’s disease, PD, amyotrophic lateral sclerosis, tuberous sclerosis, peripheral nerve injury, and depression has been reported (Ren et al., 2021). MiR-216a has been reported to regulate the progression of PD by modulating the *Bax* gene to attenuated neuronal apoptosis, and miR-216a may be a potential target for PD (Yang et al., 2020). A recent study suggested miR-8069 has the potential as an early progression biomarker for PD (Soto et al., 2023). The above studies prove the reliability of our results and the causal miRNAs in our results were worthy for further study.

This study demonstrates several strengths. Firstly, employing MR analysis allows us to emulate randomized controlled trials within observational settings, a method widely acknowledged in causal research. By utilizing an MR design, our study largely avoids the impact of reverse causation and confounding factors compared to traditional observational studies. Additionally, we conducted various sensitivity analyses, detecting no significant heterogeneity or pleiotropy. Our study represents the first attempt to employ MR analysis to investigate the causal relationship between miRNAs and PD. And provides novel biomarkers that could potentially contribute to the prevention and treatment of PD.

In this study, we conducted an MR analysis and identified two miRNAs that may have significant causal effects on the rate of cognitive decline among PD patients, and eight miRNAs nominally associated with more than three PD-related outcomes. Our findings will help to understand the pathogenesis of PD, but there are still some limitations in this study. Firstly, the sample size of miRNA eQTL data is relatively small. Although another miRNA eQTL study contained more subjects, it only identified 76 mature miRNAs that were affected by genetic variants (Huan et al., 2015). Therefore, we chose the eQTL data for 2083 miRNAs. Secondly, the participants in this study are European. Although population heterogeneity will be largely avoided, the results of our study may not be entirely applicable to subjects of other populations.

## Conclusion

In summary, we identified two miRNAs whose genetically regulated expression might have a causal role in the development of PD dementia. Our findings also provided potential miRNA biomarkers to make better and early diagnoses and risk assessments of PD.

## Data availability statement

The original contributions presented in the study are included in the article/Supplementary material, further inquiries can be directed to the corresponding author.

## Ethics statement

This research has been conducted using published studies and consortia providing publicly available summary statistics. All original studies have been approved by the corresponding ethical review board, and the participants have provided informed consent. In addition, no individual-level data were used in this study. Therefore, no new ethical review board approval was required.

## Author contributions

GS: Formal analysis, Writing – original draft. TW: Writing – original draft. XL: Data curation, Writing – original draft. DZ: Data curation, Writing – original draft. QY: Writing – original draft, Writing – review & editing. LZ: Conceptualization, Formal analysis, Writing – original draft, Writing – review & editing.

## Footnotes

^1^ http://www.phenoscanner.medschl.cam.ac.uk/

^2^ https://www.mirnet.ca/home.xhtml

## References

[ref1] Alfradique-Dunham I. von Coelln R. Blauwendraat C. Hill E. Luo L. Stillwell A.. (2021). Genome-wide association study Meta-analysis for Parkinson disease motor subtypes. Neurol. Genet. 7:e557. doi: 10.1212/NXG.0000000000000557, PMID: 33987465 PMC8112852

[ref2] Bartel D. P. J. C. (2018). Metazoan MicroRNAs. Cell 173, 20–51. doi: 10.1016/j.cell.2018.03.006, PMID: 29570994 PMC6091663

[ref3] Blauwendraat C. Iwaki H. Makarious M. B. Bandres-Ciga S. Leonard H. L. Grenn F.. (2021). Investigation of autosomal genetic sex differences in Parkinson's disease. Ann. Neurol. 90, 35–42. doi: 10.1002/ana.26090 33901317 PMC8422907

[ref4] Blauwendraat C. Nalls M. A. Singleton A. B. J. T. L. N. (2020). The genetic architecture of Parkinson's disease. Lancet Neurol. 19, 170–178. doi: 10.1016/S1474-4422(19)30287-X 31521533 PMC8972299

[ref5] Blauwendraat C. Heilbron K. Vallerga C. L. Bandres‐Ciga S. Von Coelln R. Pihlstrøm L.. (2019). Parkinson's disease age at onset genome-wide association study: defining heritability, genetic loci, and α-synuclein mechanisms. Mov. Disord. 34, 866–875. doi: 10.1002/mds.27659 30957308 PMC6579628

[ref6] Bloem B. R. Okun M. S. Klein C. J. T. L. (2021). Parkinson's disease. Lancet (North American ed) 397, 2284–2303. doi: 10.1016/S0140-6736(21)00218-X 33848468

[ref7] Boef A. G. Dekkers O. M. le Cessie S. (2015). Mendelian randomization studies: a review of the approaches used and the quality of reporting. Int. J. Epidemiol. 44, 496–511. doi: 10.1093/ije/dyv071, PMID: 25953784

[ref8] Bohush A. Niewiadomska G. Filipek A. (2018). Role of mitogen activated protein kinase signaling in Parkinson’s disease. Int. J. Mol. Sci. 19:2973. doi: 10.3390/ijms19102973, PMID: 30274251 PMC6213537

[ref9] Bowden J. Davey Smith G. Burgess S. (2015). Mendelian randomization with invalid instruments: effect estimation and bias detection through egger regression. Int. J. Epidemiol. 44, 512–525. doi: 10.1093/ije/dyv080, PMID: 26050253 PMC4469799

[ref10] Braithwaite S. P. Stock J. B. Mouradian M. M. (2012). α-Synuclein phosphorylation as a therapeutic target in Parkinson’s disease. Rev. Neurosci. 23, 191–198. doi: 10.1515/revneuro-2011-0067, PMID: 22499677

[ref11] Burgess S. Dudbridge F. Thompson S. G. (2016). Combining information on multiple instrumental variables in Mendelian randomization: comparison of allele score and summarized data methods. Wiley-Interscience 35, 1880–1906. doi: 10.1002/sim.6835, PMID: 26661904 PMC4832315

[ref12] Burgess S. Thompson S. G. (2017). Interpreting findings from Mendelian randomization using the MR-egger method. Eur. J. Epidemiol. 32, 377–389. doi: 10.1007/s10654-017-0255-x, PMID: 28527048 PMC5506233

[ref13] Chen X. Ba Y. Ma L. Cai X. Yin Y. Wang K.. (2008). Characterization of microRNAs in serum: a novel class of biomarkers for diagnosis of cancer and other diseases. Cell Discov. 18, 997–1006. doi: 10.1038/cr.2008.282 18766170

[ref14] Chen Q. Huang X. Renjie T. R. (2018). Li, lncRNA MALAT1/miR-205-5p axis regulates MPP+-induced cell apoptosis in MN9D cells by directly targeting LRRK2. Am. J. Transl. Res. 10:563, 29511451 PMC5835822

[ref15] Cho H. J. Jin S. M. Parisiadou L. Xie C. Yu J. Sun L.. (2013). MicroRNA-205 regulates the expression of Parkinson's disease-related leucine-rich repeat kinase 2 protein. Hum. Mol. Genet. 22, 608–620. doi: 10.1093/hmg/dds470, PMID: 23125283 PMC3542867

[ref16] Danborg P. B. Waldemar G. Heegaard N. H. H. Heegaard N. H. H. (2014). The potential of microRNAs as biofluid markers of neurodegenerative diseases – a systematic review. Biomarkers 19, 259–268. doi: 10.3109/1354750X.2014.904001, PMID: 24678935

[ref17] Davey Smith G. Hemani G. J. (2014). Mendelian randomization: genetic anchors for causal inference in epidemiological studies. Hum. Mol. Genet. 23, R89–R98. doi: 10.1093/hmg/ddu328, PMID: 25064373 PMC4170722

[ref18] de Gonzalo-Calvo D. Bär C. Fiedler J. Couch L. S. Brotons C. Llorente-Cortes V.. (2019). Circulating non-coding RNAs in biomarker-guided cardiovascular therapy: a novel tool for personalized medicine? Euro. Heart J. 40, 1643–1650. doi: 10.1093/eurheartj/ehy234, PMID: 29688487 PMC6528150

[ref19] Dorsey E. Sherer T. Okun M. S. Bloem B. R. (2018). The emerging evidence of the Parkinson pandemic. JPD 8, S3–S8. doi: 10.3233/JPD-181474, PMID: 30584159 PMC6311367

[ref20] Dubois B. Burn D. Goetz C. Aarsland D. Brown R. G. Broe G. A.. (2007). Diagnostic procedures for Parkinson's disease dementia: recommendations from the movement disorder society task force. Mov. Disord. 22, 2314–2324. doi: 10.1002/mds.21844, PMID: 18098298

[ref21] Feigin V. L. Nichols E. Alam T. Bannick M. S. Beghi E. Blake N.. (2019). Global, regional, and national burden of neurological disorders, 1990–2016: a systematic analysis for the Global Burden of Disease Study 2016. J. Glob. Health 18, 459–480. doi: 10.1016/S1474-4422(18)30499-X PMC6459001 30879893

[ref22] Gallo A. Alevizos I. Illei G. G. Illei G. G. (2012). The majority of MicroRNAs detectable in serum and saliva is concentrated in exosomes. Comp. Study 7:e30679. doi: 10.1371/journal.pone.0030679, PMID: 22427800 PMC3302865

[ref23] Greco M F. D. Minelli C. Sheehan N. A. Thompson J. R. (2015). Detecting pleiotropy in Mendelian randomisation studies with summary data and a continuous outcome. Stat. Med. 34, 2926–2940. doi: 10.1002/sim.6522, PMID: 25950993

[ref24] Hartwig F. P. Davey Smith G. Bowden J. (2017). Robust inference in summary data Mendelian randomization via the zero modal pleiotropy assumption. Int. J. Epidemiol. 46, 1985–1998. doi: 10.1093/ije/dyx102, PMID: 29040600 PMC5837715

[ref25] Hemani G. Tilling K. Davey Smith G. (2017). Orienting the causal relationship between imprecisely measured traits using GWAS summary data. PLoS Genet. 13:e1007081. doi: 10.1371/journal.pgen.1007081, PMID: 29149188 PMC5711033

[ref26] Huan T. Liu C. Zhang X. Tanriverdi K. Joehanes R. Chen B. H.. (2015). Genome-wide identification of microRNA expression quantitative trait loci. Nat. Commun. 6:6601. doi: 10.1038/ncomms7601, PMID: 25791433 PMC4369777

[ref27] Huang R. Yanni S. Chan K. H. K. (2020). The lung cancer associated MicroRNAs and single nucleotides polymorphisms: a Mendelian randomization analysis. In 2020 42nd annual international conference of the IEEE engineering in Medicine and Biology Society (EMBC), IEEE. 10.1109/EMBC44109.2020.9176344 33018478

[ref28] Huang H. Y. Lin Y. C. D. Li J. Huang K. Y. Shrestha S. Hong H. C.. (2020). miRTarBase 2020: updates to the experimentally validated microRNA–target interaction database. Nucleic Acids Res. 48, D148–D154, 31647101 10.1093/nar/gkz896 PMC7145596

[ref29] Jamali L. Tutunchi S. Panahi G. Borhani F. Akhavan S. Nourmohammadi P.. (2018). Circulating microRNAs as diagnostic and therapeutic biomarkers in gastric and esophageal cancers. J. Cell Physiol. 233, 8538–8550. doi: 10.1002/jcp.26850, PMID: 29923196

[ref30] Joseph C. Mangani A. S. Gupta V. Chitranshi N. Shen T. Dheer Y.. (2020). Cell cycle deficits in neurodegenerative disorders: uncovering molecular mechanisms to drive innovative therapeutic development. Aging Dis. 11, 946–966. doi: 10.14336/AD.2019.0923, PMID: 32765956 PMC7390532

[ref31] Kamat M. A. Young R. Surendran P. Burgess S. Danesh J. Butterworth A. S.. (2019). PhenoScanner V2: an expanded tool for searching human genotype–phenotype associations. Bioinfromatics 35, 4851–4853. doi: 10.1093/bioinformatics/btz469, PMID: 31233103 PMC6853652

[ref32] Li C. Song K. Gao J. Huang E. Bai Y. Liu X.. (2021). Identifying putative causal links between MicroRNAs and severe COVID-19 using Mendelian randomization. Cells 10:3504. doi: 10.3390/cells10123504, PMID: 34944012 PMC8700362

[ref33] Lin X. Gu X. L. Wang L. Shim H. Sun L. Xie C.. (2009). Leucine-rich repeat kinase 2 regulates the progression of neuropathology induced by Parkinson's-disease-related mutant α-synuclein. Neuron 64, 807–827. doi: 10.1016/j.neuron.2009.11.006, PMID: 20064389 PMC2807409

[ref34] Mens M. M. Mustafa R. Ahmadizar F. Ikram M. A. Evangelou M. Kavousi M.. (2021). MiR-139-5p is a causal biomarker for type 2 diabetes; results from genome-wide microRNA profiling and Mendelian randomization analysis in a population-based study. medRxiv. 2021:2021.05.13.21257090.

[ref35] Mu C. Dang X. Luo X.-J., (2023). Mendelian randomization reveals the causal links between microRNA and schizophrenia. J. Psychiatr. Res. 163, 372–377. 37267734 10.1016/j.jpsychires.2023.05.071

[ref36] Nalls M. A. Blauwendraat C. Vallerga C. L. Heilbron K. Bandres-Ciga S. Chang D.. (2019). Identification of novel risk loci, causal insights, and heritable risk for Parkinson's disease: a meta-analysis of genome-wide association studies. Lancet Neurol. 18, 1091–1102. doi: 10.1016/S1474-4422(19)30320-5 31701892 PMC8422160

[ref37] Nikpay M. Valsesia A. Hager J. Harper M. E. Dent R. McPherson R.. (2019). Genome-wide identification of circulating-miRNA expression quantitative trait loci reveals the role of several miRNAs in the regulation of cardiometabolic phenotypes. Cardiovasc. Res. 115, 1629–1645. doi: 10.1093/cvr/cvz030, PMID: 30715214

[ref38] O’Connor L. J. Price A. L. (2018). Distinguishing genetic correlation from causation across 52 diseases and complex traits. Nat. Genet. 50, 1728–1734. doi: 10.1038/s41588-018-0255-0, PMID: 30374074 PMC6684375

[ref39] Oliveira S. R. Correia Guedes L. Gonçalves N. Coelho M. Rosa M. M. Amaral J. D.. (2020). Circulating inflammatory miRNAs associated with Parkinson’s disease pathophysiology. Biomolecules 10:945. doi: 10.3390/biom10060945, PMID: 32585840 PMC7356527

[ref40] Ong J. S. MacGregor S. (2019). Implementing MR‐PRESSO and GCTA‐GSMR for pleiotropy assessment in Mendelian randomization studies from a practitioner's perspective. Genet. Epidemiol. Suppl. 43, 609–616. doi: 10.1002/gepi.22207, PMID: 31045282 PMC6767464

[ref41] Poewe W. Seppi K. Tanner C. M. Halliday G. M. Brundin P. Volkmann J.. (2017). Parkinson disease. Nat. Rev. Dis. Prim. 3, 1–21. doi: 10.1038/nrdp.2017.13 28332488

[ref42] Real R. Reynolds R. H. Lawton M. A. Tan M. M. X. Shoai M. Corvol J. C.. (2023). Association between theLRP1BandAPOEloci and the development of Parkinson’s disease dementia. Brain 146, 1873–1887. doi: 10.1093/brain/awac414, PMID: 36348503 PMC10151192

[ref43] Reay W. R. Kiltschewskij D. J. Geaghan M. P. Atkins J. R. Carr V. J. Green M. J.. (2022). Genetic estimates of correlation and causality between blood-based biomarkers and psychiatric disorders. Science (New York, NY) 8:eabj8969. doi: 10.1126/sciadv.abj8969 PMC8986101 35385317

[ref44] Ren F. J. Yao Y. Cai X. Y. Fang G. Y. (2021). Emerging role of MiR-192-5p in human diseases. Front. Pharmacol. 12:614068. doi: 10.3389/fphar.2021.614068 33708127 PMC7940509

[ref45] Richmond R. C. Smith G. I. M. (2022). Mendelian randomization: concepts and scope. Cold Spring Harb. Perspect. Med. 12:a040501. doi: 10.1101/cshperspect.a040501 34426474 PMC8725623

[ref46] Rizzo G. Arcuti S. Martino D. Fontana A. Logroscino G. Logroscino G. (2016). Accuracy of clinical diagnosis of Parkinson disease: a systematic review and meta-analysis. Neurology 86, 566–576. doi: 10.1212/WNL.0000000000002350 26764028

[ref47] Satterlee J. S. Jin P. Krichevsky A. Salama S. Schratt G. Wu D. Y.. (2007). Noncoding RNAs in the brain. Front. Cell Dev. Biol. 27, 11856–11859. doi: 10.1523/JNEUROSCI.3624-07.2007, PMID: 17978024 PMC6673363

[ref48] Sekula P. Pattaro C. Köttgen A. Köttgen A. (2016). Mendelian randomization as an approach to assess causality using observational data. J. Am. Soc. Neuphrol. 27, 3253–3265. doi: 10.1681/ASN.2016010098, PMID: 27486138 PMC5084898

[ref49] Shen M. Liu X. Wang Q. Li W. You X. Peng R.. (2021). Prospective study on plasma MicroRNA‐4286 and incident acute coronary syndrome. J. Am. Heart Assoc. 10:e018999. doi: 10.1161/JAHA.120.018999, PMID: 33719498 PMC8174203

[ref50] Soto M. Bravo P. Lahoz S. Garrido A. Sánchez-Rodríguez A.. (2023). Differential serum microRNAs in premotor LRRK2 G2019S carriers from Parkinson's disease. NPJ Parkinson Dis. 9:15. doi: 10.1038/s41531-023-00451-x, PMID: 36732514 PMC9894906

[ref51] Staiger D. O. Stock J. H. (1994). Instrumental variables regression with weak instruments. Mass: National Bureau of economic research Cambridge.

[ref52] Storey J. D. Tibshirani R. (2003). Statistical significance for genomewide studies. PNAS Nexus 100, 9440–9445. doi: 10.1073/pnas.1530509100, PMID: 12883005 PMC170937

[ref53] Szeto J. Y. Walton C. C. Rizos A. Martinez-Martin P. Halliday G. M. Naismith S. L.. (2020). Dementia in long-term Parkinson's disease patients: a multicentre retrospective study. Parkinson's Disease 6:2. doi: 10.1038/s41531-019-0106-4, PMID: 31934610 PMC6946687

[ref54] Tchekalarova J. Tzoneva R. (2023). Oxidative stress and aging as risk factors for Alzheimer’s disease and Parkinson’s disease: the role of the antioxidant melatonin. Int. J. Mol. Sci. 24:3022. doi: 10.3390/ijms24033022, PMID: 36769340 PMC9917989

[ref55] Tolosa E. Klein C. Rascol O. Rascol O. (2020). LRRK2 in Parkinson disease: challenges of clinical trials. Am. J. Transl. Res. 16, 97–107. doi: 10.1038/s41582-019-0301-2, PMID: 31980808

[ref56] Tolosa E. Scholz S. W. Poewe W. Poewe W. (2021). Challenges in the diagnosis of Parkinson's disease. Lancet Neurol. 20, 385–397. doi: 10.1016/S1474-4422(21)00030-2, PMID: 33894193 PMC8185633

[ref57] Wang H. Li J. Tao L. Lv L. Sun J. Zhang T.. (2022). MiR-205 regulates LRRK2 expression in dopamine neurons in Parkinson’s disease through methylation modification. Iran. J. Public Health 51, 1637–1647. doi: 10.18502/ijph.v51i7.10098, PMID: 36248294 PMC9529724

[ref58] Xue H. Shen X. Pan W. (2021). Constrained maximum likelihood-based Mendelian randomization robust to both correlated and uncorrelated pleiotropic effects. AJHG 108, 1251–1269. doi: 10.1016/j.ajhg.2021.05.014, PMID: 34214446 PMC8322939

[ref59] Yang X. Wei M. Wang A. Deng Y. Cao H. Cao H. (2020). MicroRNA-216a inhibits neuronal apoptosis in a cellular Parkinson’s disease model by targeting Bax. Metab. Brain Des. 35, 627–635. doi: 10.1007/s11011-020-00546-x, PMID: 32140823

[ref60] Zhu X. Babar A. Siedlak S. L. Yang Q. Ito G. Iwatsubo T.. (2006). LRRK2 in Parkinson's disease and dementia with Lewy bodies. Mol. Neurodegener. 1, 17–19. doi: 10.1186/1750-1326-1-17 17137507 PMC1693553

[ref61] Zimprich A. Leitner P. Lichtner P. Farrer M. Lincoln S. Kachergus J.. (2004). Mutations in LRRK2 cause autosomal-dominant parkinsonism with pleomorphic pathology. Neuron 44, 601–607. doi: 10.1016/j.neuron.2004.11.005, PMID: 15541309

